# Foreign body granuloma caused by an injection of exosomes

**DOI:** 10.1016/j.jdcr.2024.03.026

**Published:** 2024-04-27

**Authors:** Hoon Choi, Jun Ho Kwak, Bong Seok Shin, Chan Ho Na, Min Sung Kim

**Affiliations:** Department of Dermatology, Chosun University College of Medicine, Gwangju, Republic of Korea

**Keywords:** cosmetic dermatology, exosome, foreign body granuloma, rejuvenation

## Introduction

Exosomes, typically ranging between 40 and 100 nm in size, carry various biomolecules, making them potential tools for diagnostic and therapeutic applications.^1^ However, the authors encountered a case of foreign body granuloma that developed after injection of exosomes.

## Case report

**Fig 1:**
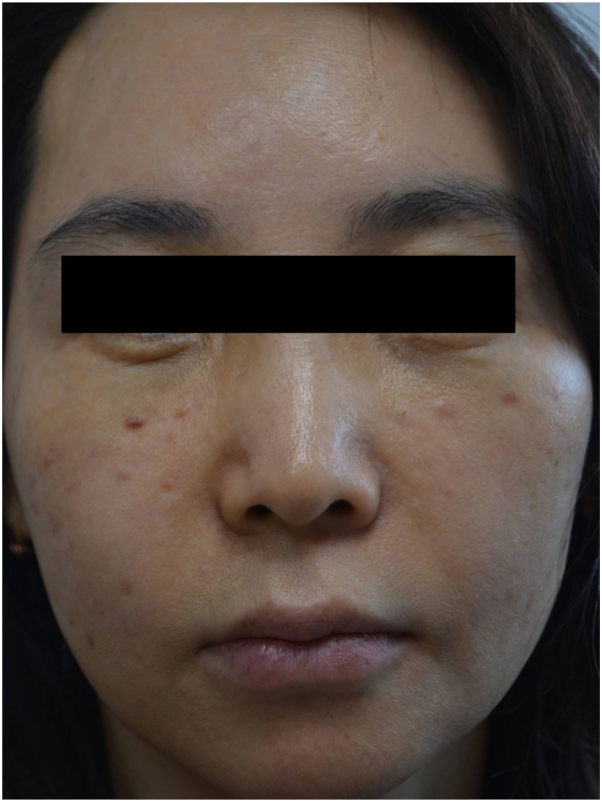
Multiple various sized flesh-colored nodules and papules on both cheeks after exosomes injection.

**Fig 2:**
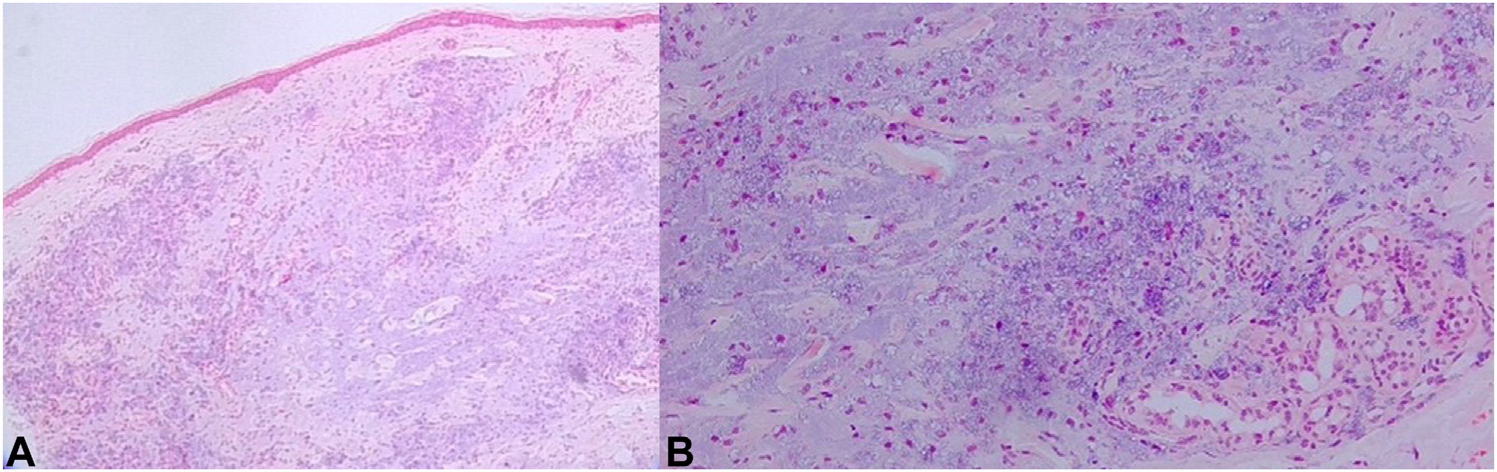
**A,** Granulomatous inflammation and amorphous basophilic material in the dermis. (H&E stain; original magnification: ×40). **B,** Histopathologic findings show granuloma mainly composed of lymphocytes and histiocytes. (H&E stain; original magnification: ×400). *H&E*, Hematoxylin and eosin.

**Fig 3:**
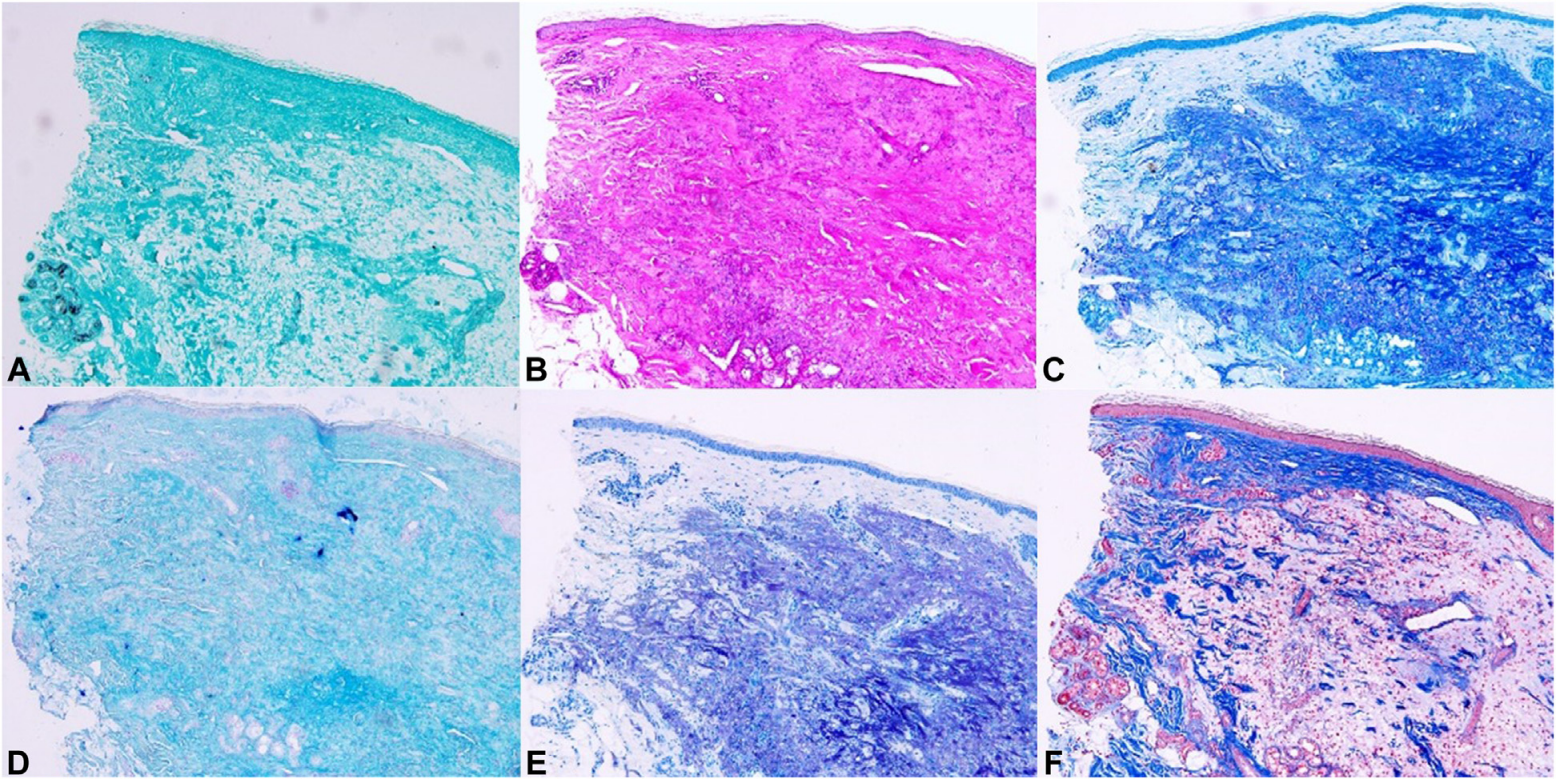
No specific findings were observed with stains such as Grocott methenamine silver (**A,** original magnification: ×200), periodic acid–Schiff (**B,** original magnification: ×200), acid-fast bacteria (**C,** original magnification: ×200), alcian blue pH 2.5 (**D,** original magnification: ×200), toluidine blue (**E,** original magnification: ×200), and Masson trichrome staining (**F,** original magnification: ×200).

A 50-year-old woman presented with multiple asymptomatic flesh-colored papules and nodules on both cheeks, which developed 7 weeks after receiving an exclusively exosome injection for wrinkle improvement and skin whitening effect at a private clinic (Fig 1). The exosome was injected intradermally with syringe into the skin on her both cheeks at spaced intervals. It was an exosome product that had not yet been officially released. Her past medical history was nonspecific, and she had no history of receiving any injections other than those exosome injection. Despite the treatment with oral doxycycline, intralesional steroid injection and hyaluronidase injection, the lesions showed little response. A 3 mm punch biopsy was performed on the lesions. The histopathologic examination revealed granulomatous reaction with histiocytic infiltration around amorphous basophilic material (Fig 2, *A*, *B*). However, no specific findings were observed with stains such as Grocott methenamine silver, periodic acid–Schiff, acid-fast bacteria, alcian blue pH 2.5, toluidine blue, and Masson trichrome staining (Fig 3, *A*-*F*). Based on the clinical presentation and biopsy results, the patient was diagnosed with foreign body granuloma most likely due to exosome injection. We prescribed oral minocycline, but the patient did not follow-up for further treatment.

## Discussion

Sun et al^2^ reported that the exosomes are well tolerated in animal models and demonstrated potential as safety of exosomes-based therapy in future. However, the effects of exosomes in animals may not be fully reflected in humans. Clinical trials in humans are 33 cases, still in the early stage and needed to evaluate about safety.^3^ There are also currently limitations in isolating, storing, and identifying exosomes.^1^

There are some exosome-based products marketed for topical cosmetic use only. These should be applied topically to the skin, and the exosomes are believed to penetrate the skin’s barrier and deliver their bioactive cargo directly to the cells in the skin. Until now, there are no exosome products registered as injectable medicines. It is important to note that injected material may be recognized as a foreign body, leading to the development of foreign body granuloma.^4^

Foreign bodies containing basophilic amorphous material can be distinguished histologically. These may include hyaluronic acid, purified polysaccharide alginate, polyacrylamide hydrogel, and metals such as aluminum.^5^ Purified polysaccharide alginate typically reacts weakly with periodic acid–Schiff and alcian blue stains but reacts strongly with toluidine blue. On the other hand, hyaluronic acid and polyacrylamide hydrogel usually give positive results with alcian blue staining. However, the alcian blue staining was negative, suggesting a different composition of these materials in this case. In addition, there was no history of exposure to aluminum, commonly used in vaccines as an adjuvant to enhance immune responses. Negative results of Grocott methenamine silver, periodic acid–Schiff, and acid-fast bacteria stains ruled out the possibility of fungal and atypical mycobacterial infections. Therefore, the histologic findings observed here are most likely a consequence of the exosome injection. The exact mechanism leading to foreign body granuloma is not fully understood, but it is possible that the granulomatous inflammation and the presence of basophilic materials seen in our case may be caused by the injection of exosomes itself or other additives mixed in the product because exosomes are often combined with other compounds such as hyaluronic acid, vitamins, and peptides.

The treatment options for complications arising from cosmetic procedures are diverse. The treatment may include intralesional corticosteroids, hyaluronidase, and empiric antibiotics such as macrolide or tetracycline.^5^ As there is no consensus on a treatment, it is important to manage these side effects appropriately based on the specific foreign body involved.

Although exosomes may hold promise as a therapeutic option, the side effects using them are not well-known. To our knowledge, there have been no reported cases of foreign body granuloma associated with exosome injection. This incident highlights the potential complications of intradermal injection of exosomes. It is important to recognize that off-label treatment can lead to adverse effects, and dermatologists should approach usage with caution.

## Conflicts of interest

None disclosed.
